# Negative effect of hepatitis in overall and progression-free survival among patients with diffuse large B-cell lymphoma

**DOI:** 10.1186/s13027-018-0190-9

**Published:** 2018-06-07

**Authors:** Mubarak M. Al-Mansour, Saif A. Alghamdi, Musab A. Alsubaie, Abdullah A. Alesa, Muhammad A. Khan

**Affiliations:** 1 Princess Noorah Oncology Center, King Abdulaziz Medical City, Ministry of National Guard Health Affairs-Western Region (WR), PO Box 9515, Jeddah, 21423 Kingdom of Saudi Arabia; 2 College of Medicine (COM), King Saud Bin Abdulaziz University for Health Sciences (KSAU-HS), Jeddah, Saudi Arabia

**Keywords:** Hepatitis B virus reactivation, Lymphoma, Diffuse large B-cell lymphoma

## Abstract

**Background:**

Hepatitis B virus (HBV) is one of the most prevalent and serious infections worldwide. HBV reactivation is a serious complication for lymphoma patients who are being treated with rituximab-containing regimen. Since the impact of HBV has not been fully evaluated on the prognosis of diffuse large B cell lymphoma (DLBCL), this study examined the effect of the hepatitis infection on the progression-free survival (PFS) and overall survival (OS) in patients with DLBCL who received rituximab-containing chemotherapy.

**Methods:**

This retrospective cohort study was conducted at Princess Noorah Oncology Center, Jeddah by reviewing all medical records of 172 DLBCL diagnosed patients and recieved Rituximab-containing chemotherapy dated from January 2009 to February 2016.

**Results:**

Out of 172 patients, 53 were found positive in hepatitis serology. The 12 of those were HBsAg-positive and 41 were HBcAb-positive. Hepatitis reactivation was observed in 1% of the patients (i.e., 2 out of 172) and both of them were HBsAg-positive. Thus, the risk of hepatitis reactivation among the HBsAg-positive patients was 17% (i.e., 2 out of 12). The predicted 3-year PFS for HBsAg-positive and HBcAb-positive were 52% (± 8%), while 76% (± 4) for HBsAg-negative and HBcAb-negative patients. On the other hand, the predicted 3-year OS for HBsAg and HBcAb-negative group is 93% (±3) while for HBsAg-positive and HBcAb-positive is 77% (±7), respectively.

**Conclusion:**

The present study demonstrated a low HBV reactivation rate of 1% exclusively in 2 patients with HBsAg-positive status diagnosed with DLBCL and receiving R-CHOP chemotherapy.

## Background

Hepatitis B virus (HBV) is one of the most prevalent and serious infections worldwide. It is estimated that more than one third of the world population has been infected with HBV and one million die annually from HBV-related liver disease [1, 2]. Therefore, HBV is considered a major health risk. HBV reactivation is a serious complication for lymphoma patients who are being treated with rituximab containing regimen [3–5]. The risk is higher for patients who are HBsAg-positive at presentation, and ranges from 26 to 53%, while the risk is approximately 2 to 20% for HBcAb-positive patients [6–8]. While the risk of reactivation and the role of prophylactic antiviral were described in the literature, nevertheless, the impact of HBsAg-positive or HBcAb-positive status on the DLBCL prognosis has not been fully evaluated. Hence, this study examined the effect of hepatitis infection on the progression-free survival (PFS) and overall survival (OS) in patients with DLBCL who received R-CHOP chemotherapy.

## Methods

This retrospective cohort study was conducted at Princess Noorah Oncology Center, Jeddah by reviewing all medical records of patients diagnosed with DBCL between January 2009 and February 2016. Patients at the age of over 16 years with histopathologically proven CD20^+^ and received frontline Rituximab-containing chemotherapy were included for this study. Patients with primary central nervous system (CNS) lymphoma, and patients who did not completed the total cycles of chemotherapy or only received palliative chemotherapy were excluded. Three reviewers conducted the case identification and data capturing using a computerized datasheet. A total of 172 patients were then identified and became eligible for this study.

### Data collected

The collected data includes patients’ demographic information, performance status, International Prognostic Index (IPI) score. The datasheet also comprised of disease characteristics, including stage, B-symptoms bone marrow involvement, and site of extranodal involvement. A complete history of the chemotherapy course and follow-up were noted as well. Patients were analyzed before and after chemotherapy for HBV serology including hepatitis B surface-antigen (HBsAg), hepatitis B envelope-antigen (HBeAg), and hepatitis B core-antibody (HBcAb IgM and HBcAb IgG). Viral load (HBV-DNA), liver profile (alanine aminotransferase [ALT], aspartate aminotransferase [AST], and total bilirubin [TB] levels) were also obtained before and after the chemotherapy. Prophylaxis to HBV with Entecavir (0.5 mg orally everyday) or Lamivudine (100 mg orally everyday) was started one week before the chemotherapy in patients with positive HBsAg and HBcAb and some patients with only positive HBcAb, and was ended 6 months after completion of the chemotherapy regimen. Date of HBV reactivation and patients post treatment status were recorded.

### Statistical method

Patients were categorized into HBsAg and/or HBcAb-negative and HBsAg-positive and/or HBcAb-positive groups. Patients characteristics and other variables were compared as follows. A student’s t-test was used for continuous variables between the two groups. A *X*^2^ test was used for a category or ordinal variables. Fisher’s exact test was used when a small sample size existed. Progression-free survival (PFS) and overall survival (OS) were estimated by using the Kaplan-Meier method [9]. PFS was defined as from the date of diagnosis to the date of relapse or the date of the last follow-up. OS was defined as from the time of diagnosis to the date of death from any cause or the date of the last follow-up. Survival curves differences were analyzed using the two-tailed log-rank test [10]. Univariate analysis by using Cox’s proportional hazards model was utilized to determine risk factors that affect PFS. Variables significantly identified in univariate analysis were tested in multivariate anvalysis by using Cox’s proportion hazard model. All statistical tests were two-sided, and a *P* value < 0.05 was considered statistically significant. Analyses were performed by use of the SPSS statistical package (IBM Corp., Released 2012, IBM SPSS Statistics for Windows, Version 21.0. Armonk, NY: IBM Corp).

### Definition of HBV reactivation

The researchers define HBV reactivation as a 10-folds or more increase in the HBV-DNA when compared to baseline during rituximab-containing chemotherapy or within 1 year after the final course of chemotherapy. Hepatitis attributable to HBV reactivation is defined as a threefold or more increase in ALT above the normal range or an increase in ALT to more than 100 U/L [11, 12].

## Results

### Patients characteristics

A total of 172 patients with biopsy proven DLBCL were identified. The median age was 58 years (18–85). Majority of patients were males (55%). In addition, 70% were diagnosed with advanced stage III-IV, and B symptoms were seen in 52%. Moreover, 77% of patients had ECOG performance status 0–2, and high-intermediate to high risk international prognostic index (IPI) was noted in 27% of patients. Extranodal involvement was reported in 58%. The most common sites of extranodal sites were liver (16%), bone (13%), lung (11%), stomach (9%), and bone marrow (9%). CNS involvement was observed in 8%. Majority of patients were treated with R-CHOP chemotherapy (89%). Patients clinical characteristics are presented in Table 1.

**Table 1:** Clinical characteristics

Characteristic	All Patients (*N* = 172)		HBsAg and HBcAb Negative (*n* = 119)		HBsAg or HBcAb Positive (*n* = 53)		*P*
	No.	%	No.	%	No.	%	
Age, years							
Median (range)	58 (18–85)		57 (18–85)		62 (32–85)		0.02
Age groups, years							
< 60	96	56	75	63	21	40	0.004
≥ 60	76	44	44	37	32	60	
Sex							0.17
Male	95	55	62	52	33	62	
Female	77	45	57	48	20	38	
Ann Arbor stage							
I	10	6	8	7	2	4	0.60
II	42	24	31	26	11	21	
III	20	12	12	10	8	15	
IV	100	58	68	57	32	60	
B symptoms							
No	82	48	58	51	24	45	0.67
Yes	90	52	61	49	29	55	
Performance status 2–4	84	49	53	45	31	58	021
Extranodal							
No	73	42	52	44	21	40	0.61
Yes	99	58	67	56	32	60	
Serum LDH > Normal International prognostic index							
Low	62	38	46	39	15	28	0.10
Low-intermediate	57	35	35	29	11	21	
High-intermediate	30	18	23	19	14	26	
High	15	9	15	13	13	24	
Liver cirrhosis							
No	170	99	118	99	52	98	0.55
Yes	2	1	1	1	1	2	
Baseline liver function							
ALT> UNL	14	8	10	8	4	8	0.78
AST > UNL	8	5	6	5	2	4	0.53
Bilirubin >UNL	6	3.5	5	4	1	2	0.39
Type of prophylaxis							
NO	132	77	119	100	13	25	
Entecavir	26	15	0	0	26	49	
Lamivudine	14	8	0	0	14	26	0.00
Chemotherapy							
R-CHOP	153	89	124	106	89	89	0.39
R-CVP	12	7	14	6	5	11	
R-CEOP	3	2	2	3	3	0	
R-EPOCH	4	2	0	4	3	8	
Consolidation radiotherapy							
Yes	17	10	12	10	5	9	0.89
No	155	90	107	90	48	91	
No. of cycles							
Median	6		6		6		0.40
Range	1–11		3–11		1–8		

### Hepatitis serology status

Fifty-three of the patients were found positive in hepatitis. The 12 of those are HBsAg-positive and 41 are HBcAb-positive patients.

The proportion of HBsAg-positive patients who were HBcAb-positive was 7% (12/172), and the proportion HBsAg-negative patients who were HBcAb-positive was 26% (41/160).

### Type of prophylaxis

At the time of the diagnosis, all HBsAg-positive patients (*n* = 12) were given antiviral therapy with either Entecavir (*n* = 9) or Lamivudine (*n* = 3). Among the 41 HBcAb-positive, 28 patients received prophylactic antiviral therapy with either Entecavir (*n* = 17) or Lamivudine (*n* = 11), and 13 patients did not receive any prophylaxis therapy.

### Hepatitis reactivation

Hepatitis reactivation was observed in 1% (2 of 172 patients). Patients who developed hepatitis reactivation were HBsAg-positive. Thus, the risk of hepatitis reactivation among the HBsAg-positive was 17% (2 of 12 patients). None of the HBcAb-positive patients developed hepatitis reactivation regardless of the prophylaxis therapy. Hepatitis reactivation occurred after R-CHOP chemotherapy in two patients. One patient received a total of 6 cycles R-CHOP chemotherapy and developed hepatitis reactivation after 6 months from the last cycle of chemotherapy, whereas the second patient received a total of 6 cycles R-CHOP chemotherapy and developed hepatitis reactivation one year after completion of R-CHOP chemotherapy. For the two patients, the only marker for HBV reactivation, which is HBV-DNA, was elevated greater than 10-folds from the baseline. None of the two patients developed hepatitis with increased liver enzymes. Both patients were on Entecavir therapy before initiation of chemotherapy. The first patient developed a relapsed disease three years after the completion of R-CHOP chemotherapy. He was treated with salvage chemotherapy ESHAP for 6 cycles, and he is in remission and alive. The second patient is in remission and alive. Both patients didn’t experience any more episodes of HBV reactivation.

### Hepatitis reactivation risk factors

Several clinical and treatment-related factors were tested to identify if theres any clinical significant difference between those who developed hepatitis reactivation (*n* = 2) and those who did not (*n* = 51), and all of them were not significant.

### Survival analysis

The median follow time was 34 months. Out of 172 patients, 50 patients (29%) had relapsed or progressive disease post primary therapy. Twenty seven patients were HBsAg-negative and HBcAb-negative, and 23 patients were either HBsAg-positive or HBcAb-positive. Out of the 23 patients, 4 patients among the HBsAg-positive had relapsed and one patient had progressive disease. Out of the remaining 18 patients with HBcAb-positive, six patients had relapsed disease and 12 patient had progressive disease. Thus, the risk of relapse or progression was 23% among the HBsAg-negative/HBcAb-negative patients (27/119). For HBsAg-positive or HBcAb-positive patients, the risk of relapse was 43% (23/53). Treatment outcomes are presented in Table 2.

**Table 2:** Treatment Outcomes

Outcome	All Patients (*N* = 172)		HBsAg/HBcAb Negative (*n* = 119)		HBsAg/HBcAb Positive (*n* = 53)		*P*
	No.	%	No.	%	No.	%	
Response							
CR/PR	122	71	92	77	30	57	0.022
PD/RD	29	17	16	14	13	24	
Disease relapse	21	12	11	9	10	19	
Predicted 3-year PFS (± SE)	67% (± 4%)		76% (± 4%)		52% (± 8%)		0.009
Death	16	9	7	6	9	17	0.21
Predicted 3-year OS (± SE)	88% (± 3%)		93% (± 3%)		77% (± 7%)		0.012

*Abbreviations*: *CR/PR* Complete response/partial response, *PD/RD* Progressive disease/refractory disease

**Fig. 1:**
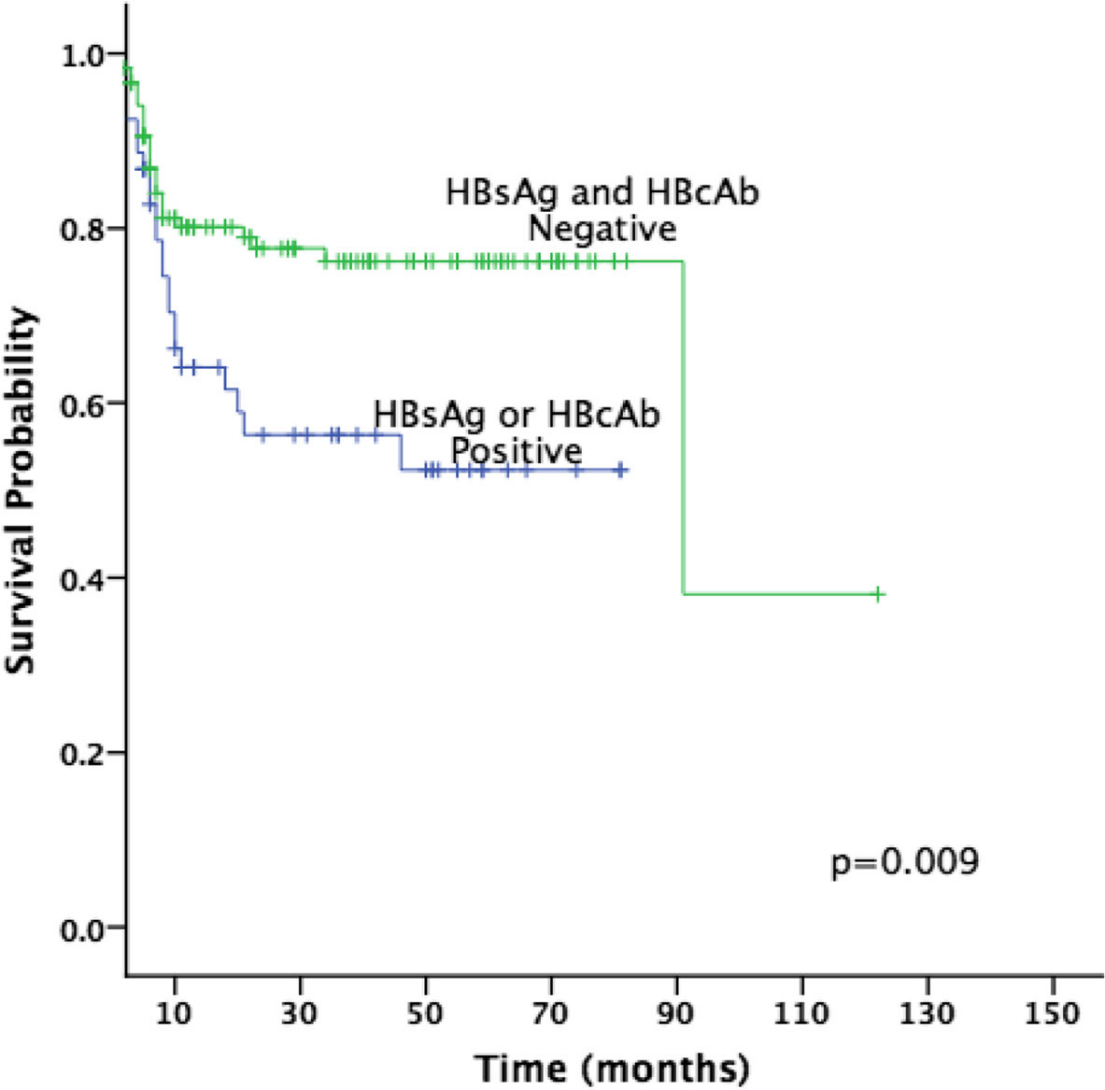
Progression-free survival of HBsAg and HBcAb negative and HBsAg or HBcAb positive groups (two-sided *p* = 0.009)

**Fig. 2:**
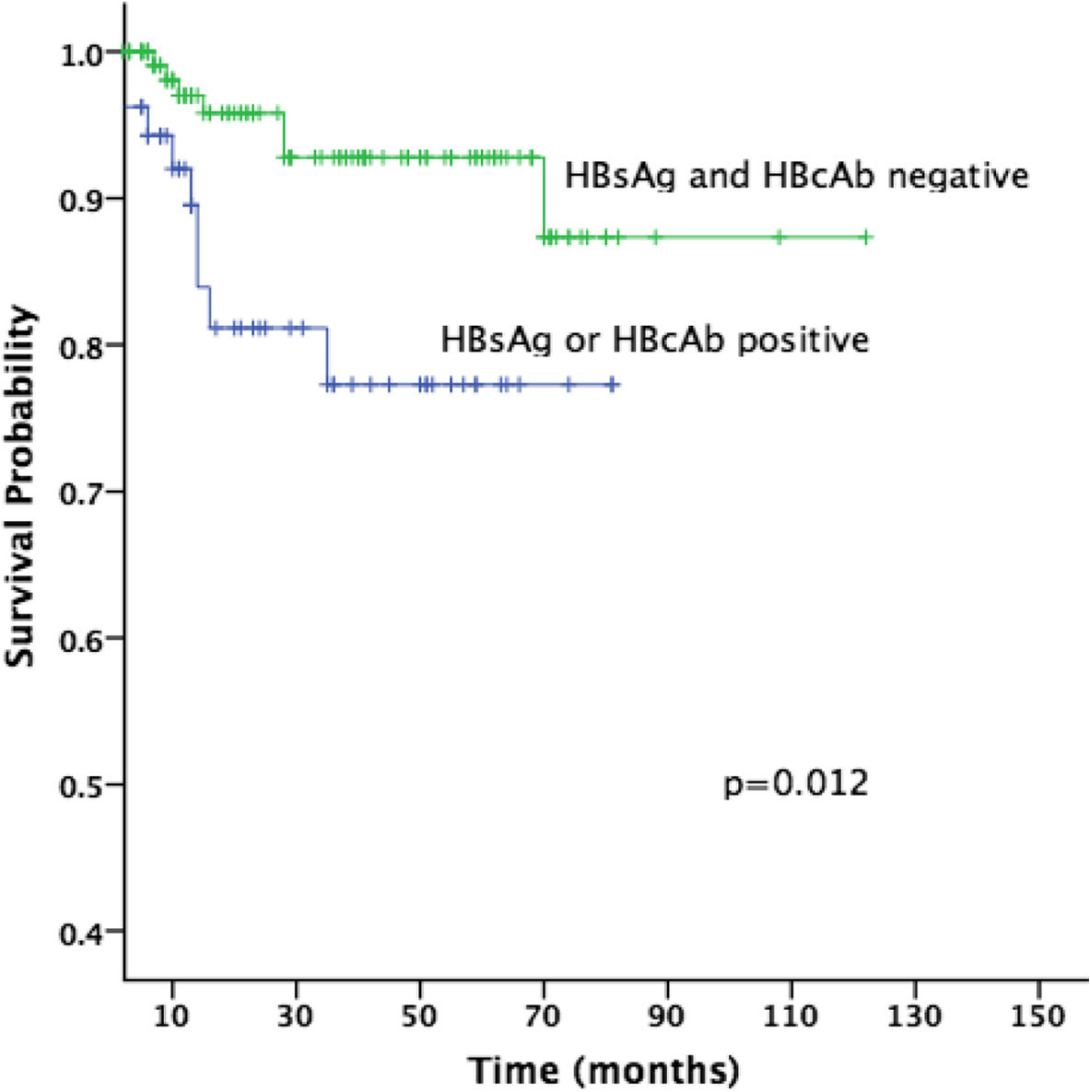
Overall survival of HBsAg and HBcAb negative and HBsAg or HBcAb positive groups (two-sided *p* = 0.012)

The median PFS for all patients was not reached with an estimated 3-year rate of 70% (± 4%). The predicted 3-year PFS for HBsAg-positive and/or HBcAb-positive were 52% (± 8%). However, the predicted 3-year PFS was 76% (± 4) for HBsAg and HBcAb-negative patients. The median OS for the whole group likewise not reached, the predicted 3-year OS was 93% (±3) and 77% (±7) for HBsAg and HBcAb-negative group and HBsAg-positive and/or HBcAb-positive group, respectively. There was significant difference in PFS (*P* = 0.009; Fig. 1) or OS (*P* = 0.012; Fig. 2) rates in HBsAg and HBcAb-positive patients as compared to HBsAg and HBcAb-negative patients, respectively. Out of all patients, 16 patients have died, 7 patients in the HBsAg-negative and HBcAb-negative group, and 9 patients in the HBsAg-positive or HBcAb-positive group (Table 2).

In univariate analysis, clinical characteristics such as stage, IPI, extranodal involvement, poor performance status and HBsAg-positive and/or HBcAb-positive status were factors significantly affected the PFS. However, in multivariate analysis, HBsAg-positive and/or HBcAb-positive and poor performance status were the two independently factors that affected the PFS (HR = 0.55; 95%CI: 0.31–0.99; *p* = 0.046) and (HR = 1.65; 95%CI: 1.09–2.49; *p* = 0.016), respectively. For OS, clinical factors such as stage, IPI, extranodal involvement, poor performance status and HBsAg-positive and/or HBcAb-positive were also found to be significant on univariate analysis. On multivariate analysis, the HBsAg-positive and/or HBcAb-positive status was the only factor independently affected the OS (HR = 0.32; 95% CI: 0.12–0.88; *p* = 0.028).

## Discussion

The prevalence of the chronic hepatitis infection (HBsAg-positive) in our patients with DLBCL was 7%. This is lower compared to other reports from China where the prevalence of the chronic hepatitis infection in DLBCL patients ranges from 8.6 to 30.2% [13].

Among our patients with HBsAg-negative, the prevalence of HBcAb-positive was 26%. Data from other countries demonstrated variable prevalence rate compared to our results. Some of those studies reported higher prevalence rate (34 to 44%) of resolved hepatitis infection in DLBCL patients, and other studies showed lower rate ranging from 2 to 20.1% [13, 14].

The risk of the hepatitis reactivation in our study was 1% for the whole group (2 out of 172). The two patients who developed hepatitis reactivation were HBsAg-positive at the time of the diagnosis with their lymphomas, and both received anti-viral treatment before initiation of the Rituximab based chemotherapy. This risk was similar to other studies in the Rituximab based therapy era. In addition, since HBV reactivation in our patients was observed 6 months to 1 year after the cessation of chemotherapy, several studies found out that cessation of rituximab-CHOP chemotherapy and antiviral such as lumivadine resulted in delayed HBV reactivation [15, 16]. Thus, prophylactic antiviral therapy was recommended to extend to at least one year after discontinuation of chemotherapy [17].

There were no patients (0%) who developed HBV reactivation in HBsAg-negative and HBcAb-positive group. This finding is consistent with the multicenter study conducted by Ji et al. [18] but in contrast with the result reported in the study of Yeo et al. (2.2 to 23.8%) [19]. The discrepancy could be explained by giving prophylactic anti-viral therapy for 68% of our patients before initiation of chemotherapy. Other explanation could be the protective immunity for the previously resolved infection, which can be confirmed by the presence of the anti-hepatitis B surface antibody (HBsAb), but unfortunately this data is missing in our study.

In this retrospective study, we demonstrated a negative impact on PFS and OS in DLBCL patients with either HBsAg-positive or HBcAb-positive serology (Figs. 1 and 2). Despite the low risk of HPV reactivation, the risk of relapse was higher in HBsAg-positive and/or HBcAb-positive group (43%) as compared to HBsAg-negative and HBcAb-negative group (23%), with a decrease in the 3-year PFS from 76 to 52%, and a decrease in the 3-year OS from 93 to 77% (Table 2). It is indeed that hepatitis infection is a significant factor that affects the survival of DLBCL patients. Although factors other than hepatitis infection were also described in literature, the exact mechanism and impact of these factors during chemotherapy remains unclear. This study, therefore, suggests to define recommendations and improvements on measures to prevent HBV reactivation among DLBCL patients.

### Limitations

One of the limitations of the study was its retrospective nature in which we reviewed the charts of the patients. Moreover, the sample size was small and some of the data was not found in patients’ charts like HBsAb which would have helped us in better result depiction.

## Conclusion

The present study demonstrated a low HBV reactivation rate of 1% exclusively in two DLBCL patients with HBsAg-positive status. We have also demonstrated that HBV has an negative impact on the PFS and the OS of DLBCL patients. HBV reactivation in DLBCL patients receiving rituximab based chemotherapy in our study was less than what is reported in the literature.

## Abbreviations

ALT: Alanine aminotransferase
ALT: Alanine Transaminae
AST: Aspartate aminotransferase
CR: Complete response
DLBCL: Diffuse Large B Cell Lymphoma
ECOG: Eastern Cooperative Oncology Group
ESHAP: Etopside, Solu-Medrol (Methylprednisolone), High-dose Ara-C (Cytarabine), Platinum (Cisplatin)
HBcAb: Hepatitis B core-antibody
HBeAg: Hepatitis B envelope-antigen
HBsAg: Hepatitis B surface-antigen
HBV: Hepatitis B virus
HBV-DNA: Hepatitis B Virus DNA
IgG: Immunoglobulin G
IgM: Immunoglobulin M
IPI: International Prognostic Index
OS: Overall survival
PD: Progressive disease
PFS: Progression free survival
PR: Partial response
R-CEOP: Rituximab,Cyclophosphamide, Etoposide, Oncovin (Vincristine) Prednisolone
R-CHOP: Rituximab, Cyclophosphamide, Hydroxydaunomycin (Doxorubicin Hydrochloride), Oncovin (vincristine), Prednisone
R-CVP: Rituximab,Cyclophosphamide, Vincristine,Prednisone
RD: Refractory disease
R-EPOCH: Rituximab, Etoposide, Prednisone, Oncovin (Vincristine), Cyclophosphamide, Hydroxydaunorubicin (Doxorubicin Hydrochloride)
TB: Total bilirubin
ULN: Upper Limit of Normal
